# Supplementary material for: Ultrafast and persistent photoinduced phase transition at room temperature monitored by streaming powder diffraction
Source: Nat Commun. 2024 Jan 24;15:267. doi: 10.1038/s41467-023-44440-3 (PMC10808240; doi:10.1038/s41467-023-44440-3)
Supplement: Supplementary file 1 — Supplementary Information [file 41467_2023_44440_MOESM1_ESM.pdf]

Supplementary Information

Ultrafast and persistent photoinduced phase transition at room temperature monitored by streaming powder diffraction

Marius Hervé[1,2], Gaël Privault[1,2], Elzbieta Trzop[1,2], Shintaro Akagi[3], Yves Watier[4], Serhane Zerdane[5], Ievgeniia Chaban[1,2], Ricardo G. Torres Ramírez[1,2], Celine Mariette[1,4], Alix Volte[4], Marco Cammarata[4], Matteo Levantino[4], Hiroko Tokoro[3,2,*], Shin-ichi Ohkoshi[6,2,*], and Eric Collet[1,2,7,*]

[1] Univ Rennes, CNRS, IPR (Institut de Physique de Rennes) - UMR 6251, 35000 Rennes, France
[2] DYNACOM IRL2015 University of Tokyo - CNRS - UR1, Department of Chemistry, 7-3-1 Hongo, Tokyo 113-0033 – Japan
[3] Department of Materials Science, Faculty of Pure and Applied Sciences, University of Tsukuba, 1-1-1 Tennodai, Tsukuba, Ibaraki 305-8577, Japan
[4] ESRF – The European Synchrotron, 71 avenue des Martyrs, CS40220, 38043 Grenoble Cedex 9, France
[5] SwissFEL, Paul Scherrer Institut, Villigen, PSI, Switzerland.
[6] Department of Chemistry, School of Science, The University of Tokyo, 7-3-1 Hongo, Bunkyo-ku, Tokyo 113-0033, Japan
[7] Institut universitaire de France (IUF)

E-mail: tokoro@ims.tsukuba.ac.jp, ohkoshi@chem.s.u-tokyo.ac.jp, eric.collet@univ-rennes.fr

Supplementary Methods 1. Synthesis and characterization of $RbMn_{0.94}Co_{0.06}Fe$
Supplementary Methods 2. Setup for Streaming Powder Diffraction
Supplementary Methods 3. X-ray Diffraction Analysis
Supplementary Methods 4. Time-dependent Rietveld Refinement of Structure
Supplementary Discussion 1. Landau model for coupled charge-transfer and symmetry-breaking
Supplementary References

Supplementary Methods 1. Synthesis and characterization of $RbMn_{0.94}Co_{0.06}Fe$

The target material was synthesized via the following method. A mixed aqueous solution of $MnCl_2{\cdot}4H_2O$ (0.094 mol.L^{-1}), $CoCl_2{\cdot}6H_2O$ (0.006 mol.L^{-1}) and RbCl (1.0 mol.L^{-1}) was reacted with a mixed aqueous solution of RbCl (1.0 mol.L^{-1}) and $K_3[Fe(CN)_6]$ (0.1 mol.L^{-1}). Elemental analysis of the prepared sample (Supplementary Table 1) shows that the formula corresponds to 93 % of $Rb_{0.94}Co_{0.06}Mn_{0.94}[Fe(CN)_6]_{0.98}{\cdot}0.2H_2O$ and 7 % of $Rb_2Mn^{II}[Fe^{II}(CN)_6]{\cdot}3.5H_2O$. The prepared compound corresponds to a dark brown powder. The Scanning Electron Microscopy (SEM) analysis revealed that the crystals are plate-shaped, with good crystallinity and average size of 0.9 ± 0.3 µm as shown in Supplementary Fig. 1a.

Supplementary Table 1. **Result of elemental analysis of $RbMn_{0.94}Co_{0.06}Fe$.**

	Rb (wt %)	Mn (wt %)	Fe (wt %)	Co (wt %)	C (wt %)	N (wt %)	H (wt %)
Calculated	23.93	14.62	15.46	0.95	19.94	23.26	0.21
Found	23.79	14.85	15.49	1.01	19.77	23.20	0.22

The temperature dependence of the molar magnetic susceptibility (χ_M) shown in Supplementary Fig. 1b indicates that the compound exhibits a thermal phase transition between the HT and LT phases with transition temperatures $T_\downarrow$ = 253 K (HT → LT) and $T_\uparrow$ = 328 K (LT → HT).

The infrared (IR) spectrum for the HT phase shows the peaks at 2153 and 2075 cm^{-1} (Supplementary Fig. 2a). The former peak is assigned to the CN stretching frequency of Mn^{II}–NC–Fe^{III} (or Co^{II}–NC–Fe^{III}) valence state of the HT phase, while the latter peak is assigned to the CN stretching frequency of Mn^{II}–NC–Fe^{II} in the impurity. Despite the small amount of impurity of only 7 %, the IR peak at 2075 cm^{-1} is observed because the ratio of the oscillator strength of the Mn^{II}–NC–Fe^{III} peak to that of the Mn^{II}–NC–Fe^{II} peak is 0.17. The IR spectrum for the LT phase is shown in Supplementary Fig. 2b. The broad peaks around 2090 cm^{-1} are mainly due to the CN stretching frequency of Mn^{III}–NC–Fe^{II} in the valence state of the LT phase with the symmetry breaking (splitting of x,z and z polar modes). This broad peak includes the peak from the impurity at 2075 cm^{-1}. The small peak observed at 2153 cm^{-1} is assigned to the CN stretching frequency of the remaining Co^{II}–NC–Fe^{III}. These results indicate a change of valence from:

- $Rb^{I}_{0.94}Co^{II}_{0.06}Mn^{II}_{0.94}[Fe^{III}(CN)_6]_{0.98}{\cdot}0.2H_2O$ for the HT phase to
- $Rb^{I}_{0.94}Co^{II}_{0.06}Mn^{III}_{0.94}[Fe^{II}(CN)_6]_{0.94}[Fe^{III}(CN)_6]_{0.04}{\cdot}0.2H_2O$ for the LT one.

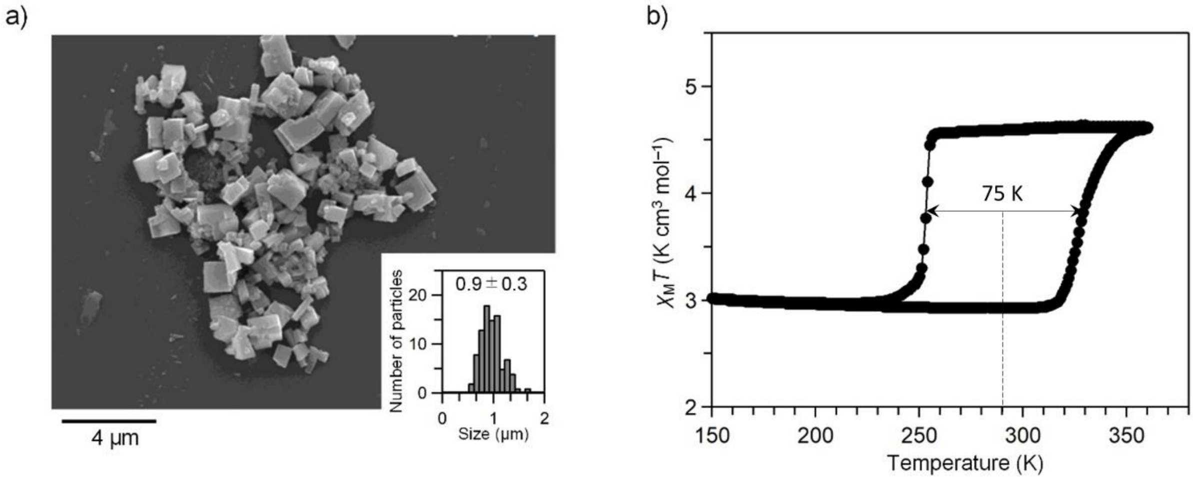

Supplementary Fig. 1. **Morphology and magnetic properties of $RbMn_{0.94}Co_{0.06}Fe$ crystals**. **a** Scanning Electron Microscopy (SEM) image and particle size distribution (inset) of the sample. **b** The $\chi_M T$ *vs T* plot measured under 5000 Oe and characterizing the charge-transfer-based phase transition between the low-temperature (LT) phase of Mn^{III}(S=2)–Fe^{II}(S=0) and the high-temperature (HT) phase of Mn^{II}(S=5/2)–Fe^{III}(S=1/2), revealing a 75 K wide thermal hysteresis ($T_{\downarrow}$ = 253 K and $T_{\uparrow}$ = 328 K), which is centred at room temperature (290.5 K).

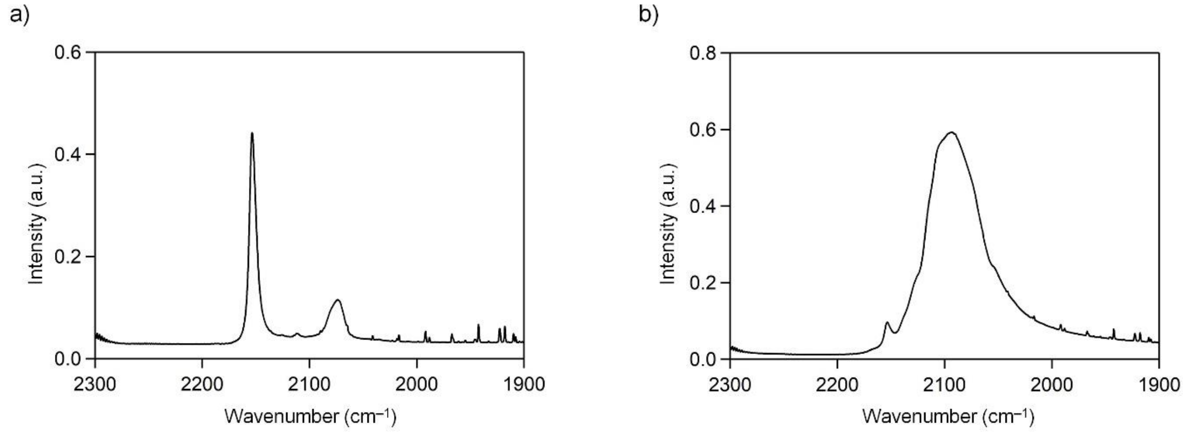

Supplementary Fig. 2. **Measured infrared spectra of $RbMn_{0.94}Co_{0.06}Fe$. a** Infrared spectrum of the high-temperature (HT) phase and **b** of the low-temperature (LT) phase. The spectrum of the HT phase was measured using a sample that was heated with a heat gun and then returned to room temperature. Similarly, the spectrum of the LT phase was measured using a sample that was cooled with liquid nitrogen and then returned to room temperature.

For the static photoinduced measurements on films, we deposited $RbMn_{0.94}Co_{0.06}Fe$ sample in pure ethanol, with a 1:90 crystal-solvent weight ratio, on a glass substrate to obtain films of the material. We cooled the sample below 180 K to reach the $Mn^{III}Fe^{II}$ LT phase and warmed it up above 350 K to reach the $Mn^{II}Fe^{III}$ HT phase. Fig. 1a shows photographs of the characteristic colour change at room temperature between the LT and HT phases. We used a 532 nm laser (FSDL-532-100T, Frankfurt Laser Company) with 100 mW output power (0.2 mm² spot size at the film position) to photoexcite during 3 s the LT phase at room temperature (centre of the thermal hysteresis). Fig. 1a shows that the HT phase is permanently photoinduced at the laser spot positions.

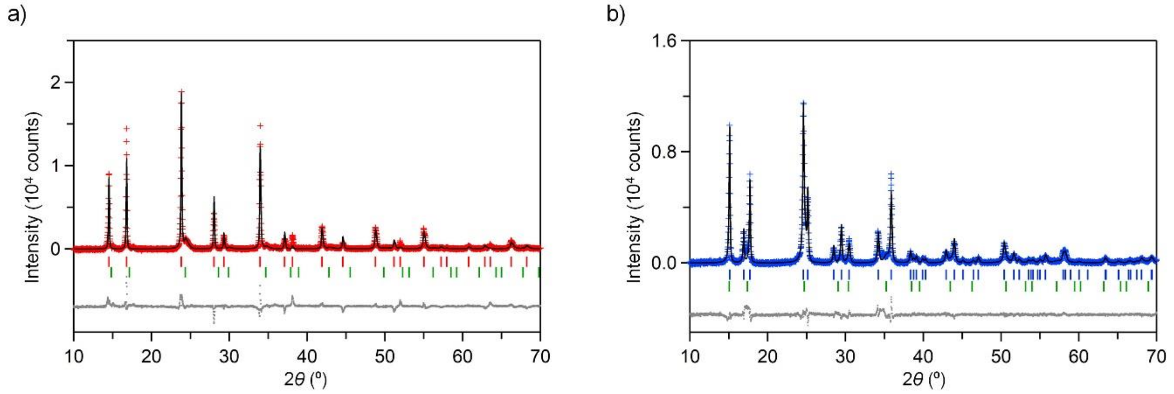

Supplementary Fig. 3. **X-ray diffraction powder pattern and Rietveld analysis of $RbMn_{0.94}Co_{0.06}Fe$.** **a** for the high-temperature (HT) phase and **b** for the low-temperature (LT) phase. The pattern for the LT phase was measured using the sample that was once cooled with liquid nitrogen and then returned to room temperature. Red or blue cross dots, black lines, and grey dots are the observed plots, calculated patterns, and their difference, respectively. Red and blue bars represent the calculated positions of the Bragg reflections in the cubic (HT phase) and tetragonal (LT phase) structures, respectively. Green bars represent the calculated positions of the Bragg reflections in the impurity of $Rb_2Mn[Fe(CN)_6]\cdot 3.5H_2O$.

The XRD powder patterns at room temperature and Rietveld analysis are shown in Supplementary Fig. 3. The structure of the HT phase is cubic ($F\bar{4}3m$) with a lattice constant of a_{HT} = 10.5495(6) Å, while that of the LT phase is tetragonal ($I\bar{4}m2$ with $a = b$ = 7.0747(8) Å and c = 10.4744(16) Å), which corresponds to the non-conventional space group $F\bar{4}2m$ with $a_{LT} = b_{LT}$ = 10.0051(11) Å and c_{LT} = 10.4744(16) Å. We also found a small signal corresponding to the passive impurity $Rb_2Mn^{II}[Fe^{II}(CN)_6]\cdot 3.5H_2O$ crystal ($Fm\bar{3}m$ a = 10.185(6) Å). The structural parameters shown in Supplementary Table 2 are characteristic of the $Mn^{II}Fe^{III}$ HT phase and $Mn^{III}Fe^{II}$ LT phase with the symmetry-breaking Jahn-Teller distortion, which stabilizes the LT Mn^{III} state. The unit cell volumes (V_{HT} = 1174 Å^3 for the HT $Mn^{II}Fe^{III}$ phase and V_{LT} = 1048 Å^3 for the LT $Mn^{III}Fe^{II}$ phase) are smaller than the ones of the pure $RbMn[Fe(CN)_6]$ system (V_{HT} = 1178 Å^3 and V_{LT} = 1055 Å^3),[1] which indicates that the Co doping results in a chemical pressure favouring the lower volume LT phase. Compared to the pure $RbMn[Fe(CN)_6]$ material exhibiting a thermal hysteresis centred at 267 K ($T_{\downarrow}$ = 231 K and $T_{\uparrow}$ = 304 K), the Co doping in $RbMn_{0.94}Co_{0.06}Fe$ allows for centring the 75 K wide regime of bistability at room temperature (290.5 K).

CCDC #2259400 (LT) and #2259401 (HT) contain the supplementary crystallographic data for this paper. These data can be obtained free of charge from The Cambridge Crystallographic Data Centre via www.ccdc.cam.ac.uk/data_request/cif.

Supplementary Methods 2. Setup for Streaming Powder Diffraction

Time-resolved X-ray diffraction (TR-XRD) measurements were performed on beamline ID09 at the European Synchrotron Radiation Facility (ESRF). The ID09 setup for ultrafast X-ray diffraction has been described in detail previously.[2] The experiment was performed while the ESRF storage ring was operated in 7/8+1 filling mode with a single bunch current of 4 mA, corresponding to an X-ray pulse duration of ~35 ps (HWHM). The X-ray central energy was 14.963 keV (λ = 0.824 Å, 2 % bandwidth), and the beam was focused to a spot size of 25×25 μm^2 (FWHM) at the sample position. To perform laser pump – X-ray probe measurements, isolated X-ray pulses are synchronized with a 1 ps laser, whose spot size was 210 µm×250 µm (V×H, FWHM). The larger laser spot size ensures a homogeneous excitation of the sample in the volume probed by the X-rays. The laser wavelength, set to 650 nm, excites crystals on the red edge of their absorption spectrum, with a penetration depth of 1.9 µm,[3] larger than the average size of the crystals (0.9 ± 0.3 µm). Additionally, we studied how photo-response changes with laser fluence, which was varied between 0 and 150 $mJ.cm^{-2}$. The pump-probe measurement was performed at 1 kHz repetition rate.

For the present streaming powder diffraction study, the $RbMn_{0.94}Co_{0.06}Fe$ crystals were dispersed in ethanol, and the solution flowed through a free-flowing liquid jet and interact with laser and X-ray pulses. The jet size was 300 µm, and the crystal-solvent weight ratio was 1:270, which corresponds to ~500 crystals per shot in the X-ray focus. The jet velocity was set to 5 $m.s^{-1}$ at the interaction region, which corresponds to a flow rate of 1.2 $L.h^{-1}$, *i.e.* 0.33 $mm^3.ms^{-1}$. Given the repetition rate and laser spot size, the laser beam irradiates a volume of 0.016 mm^3 (210 µm×250 µm×300 µm) every 1 ms. Thus, among the solution that flow through the jet nozzle, only a fraction of 5 % is irradiated by the laser. Additionally, a single photoexcited volume moves 5 mm downstream before the next laser shot arrives (1 ms later), which is lower than the vertical laser spot size (210 µm). This ensures that a renewed ensemble of fresh crystals in the ground state, which was not excited by the previous laser shot, is interacting with each laser pump – X-ray probe pulses duo.

The chosen flow rate also limits the observation time window for pump-probe measurements. Indeed, the jet velocity imposes that the volume irradiated by the laser pump beam moves downstream with the jet flow. This is depicted on Supplementary Fig. 4a, where snapshots of the interaction area are represented at different times after laser excitation. While the overlap between X-ray and laser beams is set at time 0, the excitation density created by the laser moves downstream on the µs timescale compared to the X-ray probed volume, therefore decreasing and eventually losing initial overlap. To determine experimentally the associated time window, we measured time-dependent XRD patterns for $RbMn_{0.94}Co_{0.06}Fe$ on the [0.1 µs; 800 µs] timescale, in the case of high fluence (62 $mJ.cm^{-2}$, Supplementary Fig. 4b). In this case, it is known that the created photoinduced cubic phase (PIC) is stable, and we showed in the main text that is formed within 100 ps. We thus checked the time evolution of the Bragg peaks associated to this phase (Supplementary Fig. 4c): the intensity of $(400)_{PIC}$ peak is constant up to 10 µs, and rapidly drops for longer times. This is due to the loss of overlap between the initially photoexcited region and the X-ray probed volume as the jet flows downstream: crystals that are initially photoconverted are not in the probed volume

anymore. The measured time window of 10 µs can be rationalized by considering the theoretical evolution of laser – X-ray overlap as the jet flows. Assuming that the initial excitation profile is proportional to the laser intensity $I_{\mathrm{L}}(z) \propto \exp\left(-\frac{4\ln 2.z^2}{\mathrm{FWHM}_{\mathrm{L}}^2}\right)$ and that it flows downstream with a central value of $z_{\mathrm{L}}(t) = v_{\mathrm{jet}}.t$, the diffraction signal at a specific delay t will be proportional to the laser – X-ray overlap:

$$\Delta I(t) \propto \int_z I_{\mathrm{X}}(z).I_{\mathrm{L}}(z,t)dz \propto \int_z \exp\left(-\frac{4\ln 2.z^2}{\mathrm{FWHM}_{\mathrm{X}}^2}\right).\exp\left(-\frac{4\ln 2.\left(z-z_{\mathrm{L}}(t)\right)^2}{\mathrm{FWHM}_{\mathrm{L}}^2}\right)dz \qquad (1)$$

i.e.,

$$\Delta I(t) \propto \exp\left(-\frac{4\ln 2.z_{\mathrm{L}}(t)^2}{\left(\mathrm{FWHM}_{\mathrm{X}}^2+\mathrm{FWHM}_{\mathrm{L}}^2\right)}\right) \qquad (2)$$

The modelled overlap, calculated using experimental parameters ($\mathrm{FWHM}_{\mathrm{X}}$ = 25µm; $\mathrm{FWHM}_{\mathrm{L}}$ = 210µm; v_{jet} = 5 m.s^{-1}), is displayed in Supplementary Fig. 4c, and shows very good agreement with experimental data. We thus considered 10 µs as the maximum observation time window for the streaming powder setup, and the model shows that it corresponds to a loss in overlap of ca. 10 % compared to time 0.

TR-XRD measurements usually require long data acquisitions (> 1 h) for getting good data quality. To accommodate it, jet was circulated in closed loop, and accordingly the part of the solution that is partially excited once was reused. In order to compensate for the non-reversible photo-conversion of the already-excited crystals, a cooling device was inserted in the liquid circulation loop. It consists of a 1.5 m-long heat exchanger between the solution and a flow of cooled nitrogen able to bring the solution temperature at –45°C. The cooling temperature, measured using a thermocouple at the end of the heat exchanger, was controlled using a PID feedback loop on the flow rate of cooling nitrogen gas. Following passage through the cooling device, the solution flows through a 2 m tube at room temperature before the jet nozzle, ensuring that the solution is brought back to room temperature at the interaction region. This was checked by measuring the temperature of the solution at the jet position, using a thermocouple. Additionally, temperature of the PBA reservoir was also checked from times to times. By cooling the sample, crystals photo-converted by light excitation are thermally brought back to the LT phase below the hysteresis regime. This device is compulsory as at high laser fluence a fraction of 5 % of the solution flowing through the jet is converted. Given the flow rate and a global volume of solution used in the closed loop of typically 100 mL, the whole volume passes ~12 times per hour through the nozzle, and therefore a conversion of ~50 % within 1 hour would be observed without any cooling device. When using the cooling device, such a drift was not observed over hours, and long acquisitions were thus possible using this configuration.

X-ray diffraction patterns were recorded in transmission geometry using a Rayonix MX170-HS CCD detector with an integration time of 5 s for each image. The delay between the laser pump pulse and the X-ray probe pulse was varied from –3 ns to +10 µs, and we collected typically 20 diffraction images for each delay. As crystals are randomly oriented in the liquid jet, diffraction images consisted of rings that were azimuthally integrated using pyFAI.[4] We normalized each image over the Q-range of [5.5; 6.0] Å^{-1}, where diffraction signal remains constant along the dynamics.

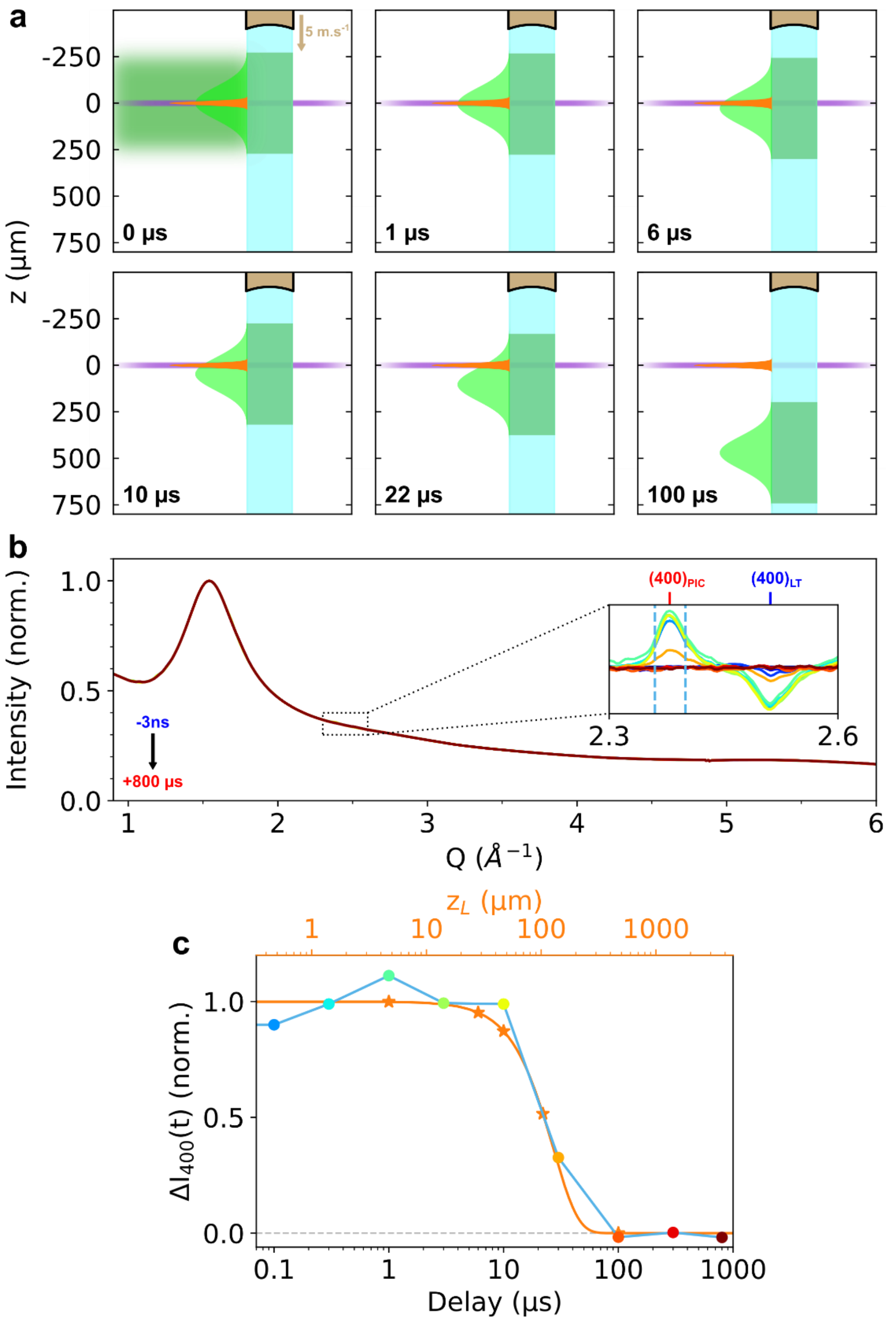

Supplementary Fig. 4. **Observation time window in streaming powder diffraction.** **a** Schematic representation of excitation profile at different delays, showing the overlap between the laser-irradiated volume (green gaussian profile, created by laser laser irradiation at 0 µs) and the X-ray probed volume (orange gaussian profile) in the jet, calculated using the experimental conditions. **b** Diffraction patterns measured for a solution of $RbMn_{0.94}Co_{0.06}Fe$ at different delays (from -3 ns, blue curve, to +800 µs, red curve, see panel **c** for the correspondence between colours and delays) after laser excitation at 62 mJ.cm^{-2}. The inset shows a zoom around the (400) Bragg peaks associated with the low-temperature (LT) and photoinduced cubic (PIC) phases, where the differential signal with respect to -3 ns is shown. It shows a positive and constant signal around $(400)_{PIC}$ before 10 µs, due to photo-transformation of the crystals, that vanishes after 10 µs, because of loss in laser – X-ray overlap. **c** Integrated intensity of $(400)_{PIC}$ peak as a function of delay (color-coded dots and blue curve, integrated over the dashed lines in the inset of **b**), together with the modelled laser – X-ray overlap (orange curve). Orange star markers correspond to delays at which the snapshots are represented in panel **a** (1 µs: 0.1 % loss of overlap, 6 µs: 5 % loss, 10 µs: 10 % loss, 22 µs: 50 % loss, 100 µs: 100 % loss), and orange axis represents the corresponding central position of the photo-excited volume along the jet, z_L.

Supplementary Methods 3. X-ray Diffraction Analysis

The azimuthally integrated X-ray scattering patterns contain two different contributions: the broad scattering from the solvent (ethanol) and sharper peaks from the crystal suspension. The former contribution dominates the pattern due the low crystal-solvent weight ratio (1:270). Supplementary Fig. 5a compares the typical diffraction pattern of crystals of $RbMn_{0.94}Co_{0.06}Fe$ in the LT phase dispersed in ethanol, with the pattern of pure ethanol. In order to extract the diffraction pattern of crystals of $RbMn_{0.94}Co_{0.06}Fe$ only, the solvent contribution was initially subtracted from the total signal. Supplementary Fig. 3b shows the result of this initial subtraction, where peaks from crystals in the LT phase appear more clearly on top of a modulated background. Residual background was then fitted using a series of three different smooth polynomials shown in Supplementary Fig. 5b for Q-ranges [0.9; 1.5] $Å^{-1}$ (order 10, yellow), [1.5; 1.9] $Å^{-1}$ (order 7, blue) and [1.9; 6.0] $Å^{-1}$ (order 8, magenta). The resulting X-ray diffraction pattern of $RbMn_{0.94}Co_{0.06}Fe$ is reported in Supplementary Fig. 5c.

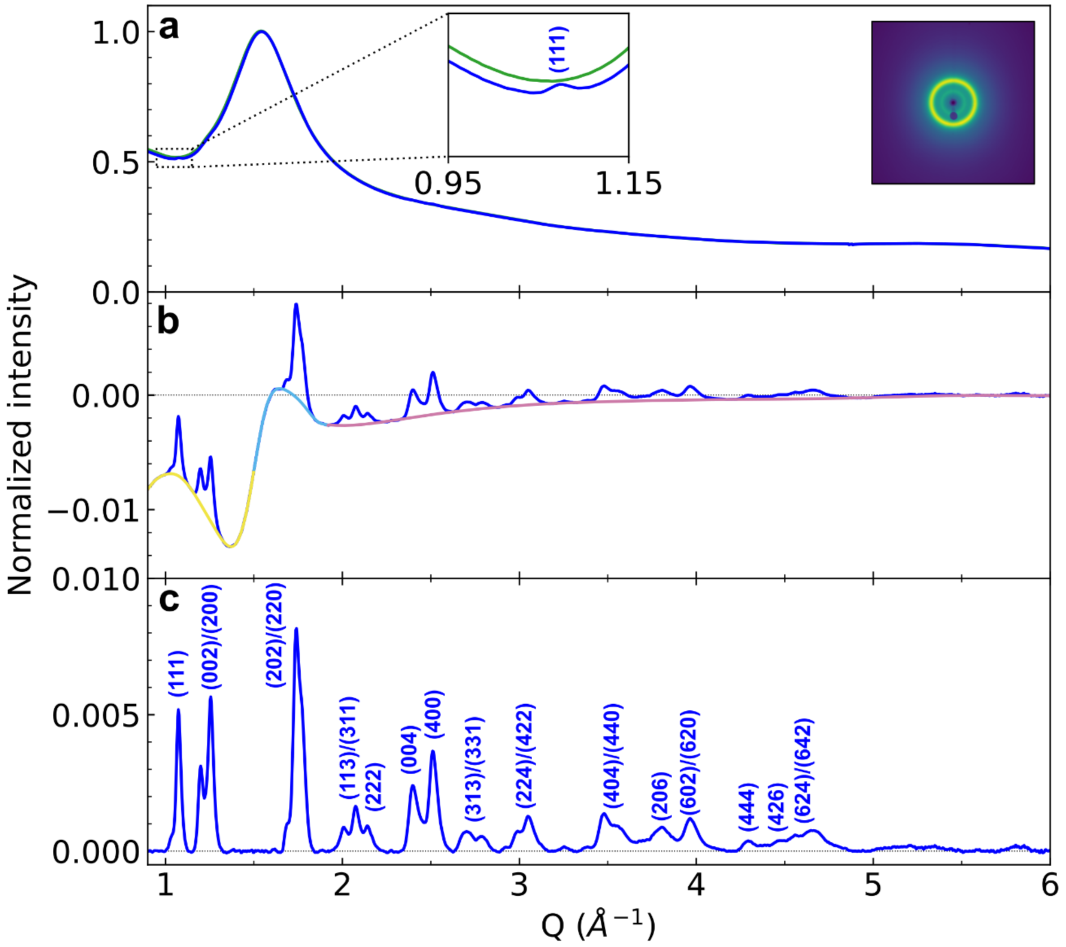

Supplementary Fig. 5. **X-ray scattering from powder streaming. a** Diffraction pattern measured for low-temperature-phase solution of $RbMn_{0.94}Co_{0.06}Fe$ (LT, blue), compared with the diffraction pattern of pure ethanol (green). The inset is a zoom between 0.95 $Å^{-1}$ and 1.15 $Å^{-1}$, that corresponds to the region of (111) Bragg peak of the crystals. Raw diffraction image, obtained for 5 s integration time, is also shown. **b** Difference between the diffraction pattern of $RbMn_{0.94}Co_{0.06}Fe$ and pure ethanol (blue curve), done as a first step in background subtraction. Result of polynomial background fit is plotted in yellow (degree: 10), blue (degree: 7), magenta (degree: 8). **c** Diffraction pattern of LT-phase crystals of $RbMn_{0.94}Co_{0.06}Fe$ after background subtraction. (hkl) Miller indices of the LT phase are indicated in the $F\bar{4}2m$ space group.

To validate the streaming powder diffraction methodology, Supplementary Fig. 6 compares the collected diffraction patterns to the ones from powder diffraction shown in Supplementary Fig. 3, for both LT (blue) and HT (red) phases. The agreement is very good considering that the data from Supplementary Fig. 3 were collected using a monochromator, while the time-resolved data were collected in polychromatic mode, which broadens Bragg peaks. Nonetheless, the essential features of the changes in diffraction patterns between HT and LT phases are observed, such as the splitting of Bragg peaks along scattering vector Q (e.g., (400) and (004) around 2.5 Å^{-1} are equivalent in the HT phase and split in the LT phase due to symmetry breaking) and their shifting to higher Q due to lattice contraction (Supplementary Fig. 6). The present static measurements therefore confirm the capability to record diffraction patterns of dispersed crystals using streaming powder diffraction.

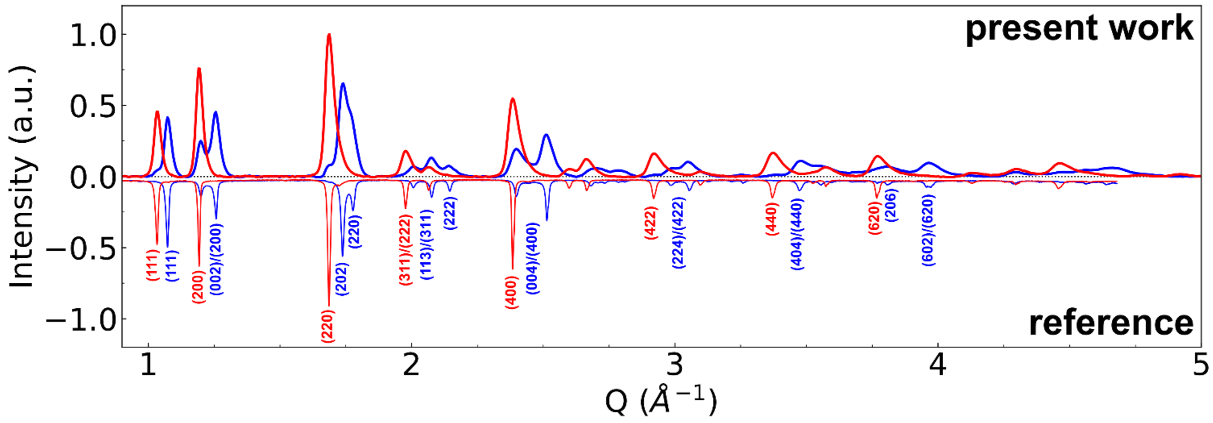

Supplementary Fig. 6. **Diffraction patterns from streaming powder**. Data from low temperature (LT in blue, up) or high temperature (HT in red, up) phases are compared with reference powder patterns measured with monochromator (below). Miller indices of the two phases are indicated in the $F\bar{4}2m$ for LT and $F\bar{4}3m$ HT space groups.

For the Rietveld refinement of the X-ray diffraction data, we used the TOPAS-Academic version 6 software,[5] with a fixed wavelength of 0.827389 Å^{-1} (15 keV as calibrated at the ID09 ESRF beamline). Supplementary Fig. 7 shows the result of refinement for the pure LT and HT phases over the scattering vector Q-range [0.9; 5.0] Å^{-1} using the structures found from powder diffraction (Supplementary Fig. 3). For both phases, lattice parameters were set free, and refinement also included fitting of isotropic Debye-Waller thermal factors of all the atoms. Given the symmetry change between tetragonal LT phase and cubic HT phase, refinement was done considering space group $F\bar{4}2m$ for LT phase and $F\bar{4}3m$ for HT phase in order to directly compare both phases, as explained in our recent work.[1] Sometimes a weak residual HT phase was observed in the equilibrium X-ray diffraction data and did not evolve during the few hours necessary for time scan acquisition. The lattice parameters of the two phases obtained from refinement are: a_{HT} = 10.544(2) Å for the HT phase with R_{wp} = 15 %, and a_{LT} = 10.023(2) Å, c_{LT} = 10.461(2) Å for the LT phase with R_{wp} = 11 %. Here, the uncertainties correspond to the standard deviation of the associated Rietveld fit. Given the 2 % bandwidth of the synchrotron X-ray beam, those values agree very well with the powder X-ray diffraction measurements performed in our laboratory with monochromator

(Supplementary Fig. 3) and confirm the accuracy of the technique (see Supplementary Table 2 for the full comparison).

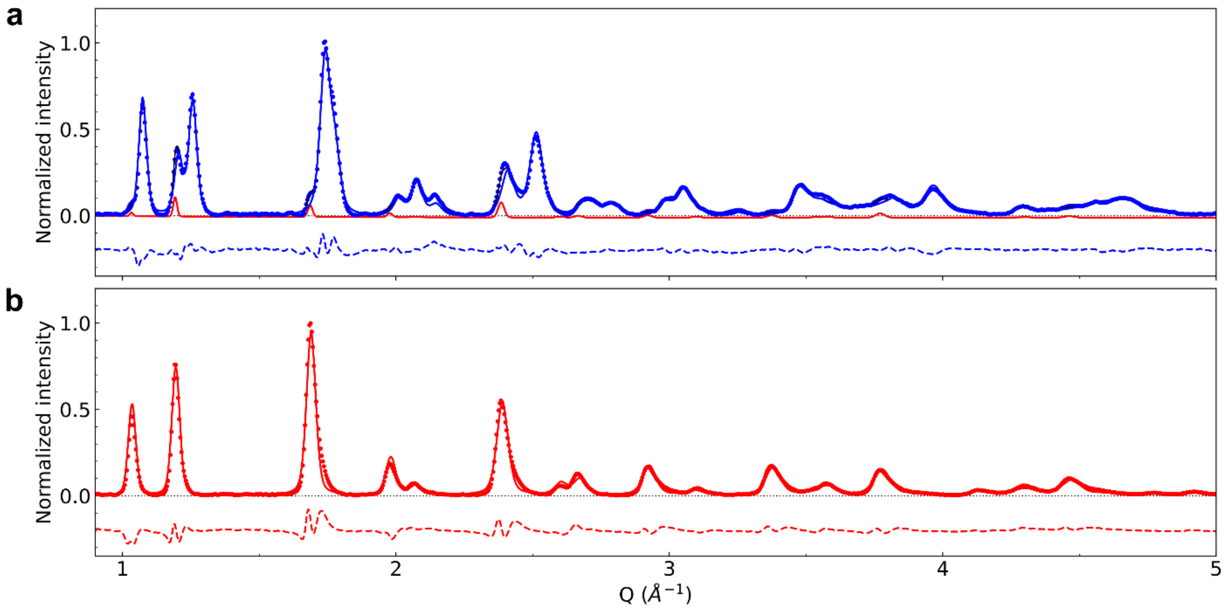

Supplementary Fig. 7. **Rietveld refinement of streaming powder diffraction data**. **a** for the low-temperature (LT) branch at room temperature. **b** for the high-temperature (HT) branch. Dots represent the raw data, and the residual of the fit is displayed as the dashed line (vertically shifted for clarity). For LT solution, the global fit (dark line, R_{wp} = 11 %) includes the contribution of LT phase (blue line, x = 93 %) and residual HT phase (red line, x = 7 %). For data collected in the HT branch, the fit used a single HT phase (red line, R_{wp} = 15 %).

Supplementary Table 2. **Structural data for the measured phases.** Low-temperature (LT) and high-temperature (HT) data were measured with conventional powder diffraction and streaming powder diffraction, and are compared with the structural data of the photoexcited tetragonal (PT) and photoinduced cubic (PIC) phases, at a delay of 1 ns and for a fluence of 36 mJ.cm^{-2}. The uncertainties (in brackets) correspond to the standard deviation of the associated Rietveld fit.

phase		LT Conventional powder diffraction	HT Conventional powder diffraction	LT Streaming powder diffraction	HT Streaming powder diffraction	PT @ 1 ns Streaming powder diffraction	PIC @ 1 ns Streaming powder diffraction
space group		$F\bar{4}2m$	$F\bar{4}3m$	$F\bar{4}2m$	$F\bar{4}3m$	$F\bar{4}2m$	$F\bar{4}3m$
a (Å)		10.0051(11)	10.5495(6)	10.023(2)	10.544(2)	10.075(7)	10.561(5)
b (Å)		10.0051(11)	10.5495(6)	10.023(2)	10.544(2)	10.075(7)	10.561(5)
c (Å)		10.4744(16)	10.5495(6)	10.461(2)	10.544(2)	10.464(8)	10.561(5)
V (Å^3)		1048.5(3)	1174.1(1)	1051.0(5)	1172.3(5)	1062(3)	1178(2)
η		0.0469(4)	0	0.0480(4)	0	0.043(2)	0
v_s		-0.1069(8)	0	-0.1035(8)	0	-0.094(3)	0
Mn–N (Å)	*x*	1.9870(3)	2.1626(2)	1.9906(3)	2.1615(3)	2.001(2)	2.165(1)
	y	1.9870(3)	2.1626(2)	1.9906(3)	2.1615(3)	2.001(2)	2.165(1)
	z	2.2551(3)	2.1626(2)	2.2523(5)	2.1615(3)	2.253(2)	2.165(1)
<Mn–N> (Å)		2.0764(3)	2.1626(2)	2.0778(3)	2.1615(3)	2.085(2)	2.165(1)
Fe–C (Å)	*x*	1.8830(3)	1.9981(2)	1.8863(3)	1.9970(3)	1.896(2)	2.0002(9)
	y	1.8830(3)	1.9981(2)	1.8863(3)	1.9970(3)	1.896(2)	2.0002(9)
	z	1.8205(3)	1.9981(2)	1.8182(4)	1.9970(3)	1.819(2)	2.0002(9)
<Fe–C> (Å)		1.8622(3)	1.9981(2)	1.8636(3)	1.9970(3)	1.870(2)	2.0002(9)

Supplementary Methods 4. Time-dependent Rietveld Refinement of Structure

Analysis of time-resolved and fluence-dependent diffraction patterns was performed following the same procedure as for pure phases. Background subtraction and Rietveld refinement were performed at each delay. In this case, Rietveld refinement was done with different phases: a photoexcited tetragonal $F\bar{4}2m$ phase (PT), describing the evolution of the initial phase and a photo-induced cubic $F\bar{4}3m$ phase (PIC), describing the growing phase. A residual signal from HT phase (HT_{res}) was sometimes observed at negative delay, included in Rietveld refinement and then kept constant at positive delays, as it is optically inactive. For each phase, we used the model resulting from the refinement of pure (static) LT and HT phases, with fixed isotropic Debye-Waller thermal factors. At negative delays, refinement included only the LT and HT_{res} phases, while the three phases (PT, PIC and HT_{res}) were included at positive delays. Supplementary Fig. 8 shows typical results of refinement, at -3 ns and +1 ns, together with the contribution of the different phases. At -3 ns, when diffraction is measured before laser excitation, the ground LT lattice parameters are (a_{LT} = 10.008(3) Å, c_{LT} = 10.456(4) Å). At +1 ns the LT phase has evolved towards PT lattice parameters of (a_{LT} = 10.050(7) Å, c_{LT} = 10.438(8) Å), while some PIC phase has appeared with a phase fraction increased to 37 % (a_{PIC} = 10.534(5) Å). For each time delay, the initial conditions of Rietveld refinement consisted of the result of refinement at the preceding delay (sequential refinement). Phase fractions are refined for all the phases (PIC phase fraction, x_{PIC}, and PT phase fraction, $1\text{-}x_{PIC}$), together with the corresponding lattice parameters, a_{PT}, c_{PT}, a_{PIC}, if the associated phase fraction is above 20 %. For all delays, we calculated the volume of each phase as well as the symmetry-breaking order parameter:

$$V_{PT} = a_{PT}^2 . c_{PT}, \quad V_{PIC} = a_{PIC}^3 \quad \text{and} \quad \eta = \frac{2}{\sqrt{3}}\left(\frac{c_{PT}-a_{PT}}{a_{HT}}\right) \tag{3}$$

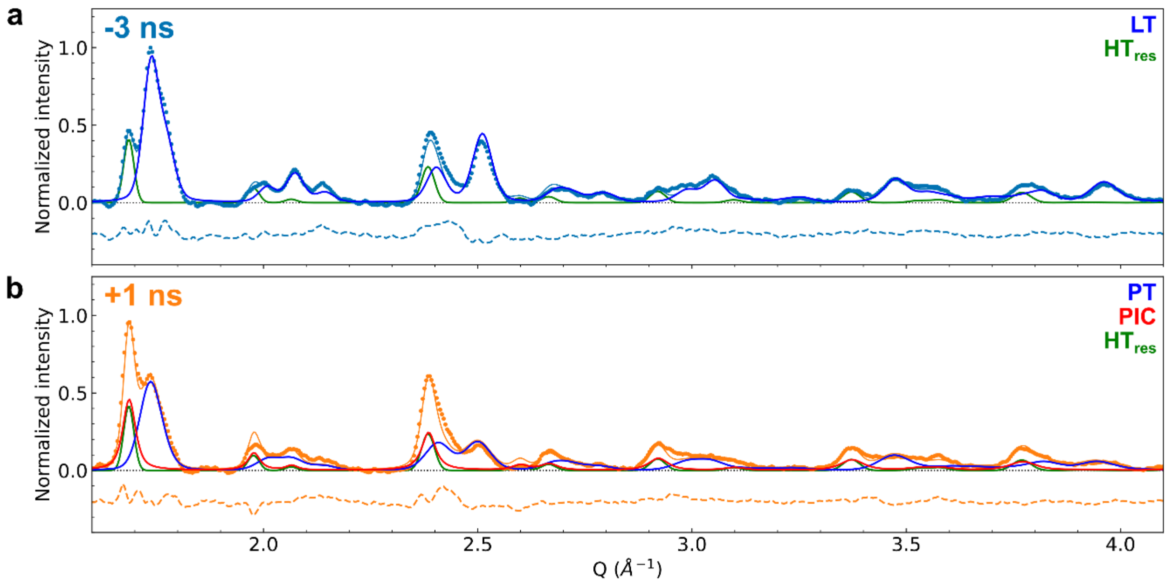

Supplementary Fig. 8. **Examples of Rietveld refinement.** Data obtained at two different delays, -3 ns (**a**), +1 ns (**b**), for a laser fluence of 36 mJ.cm^{-2}. For each delay, dots represent the raw data together with the full fit (line of same colour as dots), and the residual of the fit (dashed line, shifted by -0.2). The contribution of the 3 phases is also shown: low-temperature (LT) or photoexcited tetragonal (PT) phase (blue line), photo-induced cubic phase (PIC, red line), and residual high-temperature HT_{res} phase (green line). R_{wp} value is 14 % at both delays.

As explained in the main text, we extracted the Mn-N distances for the PT and PIC phases, from Rietveld refinement. Fig. 3b of the main text displays the bond lengths along both axes (Mn-N_{xy} and Mn-N_z), calculated from the refined lattice parameters with fixed atomic coordinates in the structure refinement. Alternatively, we also refined the Mn-N distances in the structural model of the PT phase, as shown in Supplementary Fig. 9 for 36 mJ.cm^{-2} fluence. To avoid overparameterization, the atomic coordinates of N and C atoms were refined keeping C-N bonds between 1.10 Å and 1.17 Å. The Mn-N distances obtained at negative delays in this way are shifted from their expected values (1.53 Å instead of 2.00 Å for Mn-N_{xy}, and 2.36 Å instead of 2.25 Å for Mn-N_z). However, the time-resolved signal obtained in this way shows a trend similar to that in Fig. 3b of main text: at positive delays Mn-N_{xy} increases while Mn-N_z decreases. This shows that refinement of atomic coordinates gives the good relative trend, despite wrong absolute values probably due to Co doping, directly evidencing the reverse Jahn-Teller distortion towards more cubic symmetry.

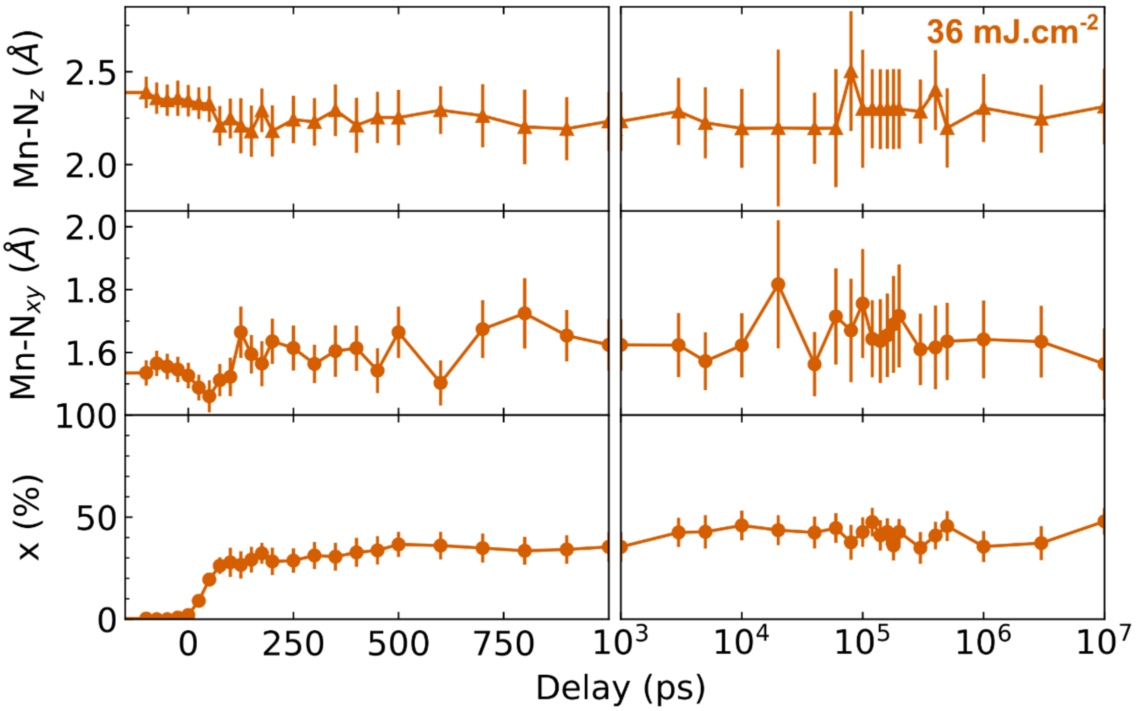

Supplementary Fig. 9. **Results from the Rietveld fitting.** The refinement of atomic coordinates provides the temporal evolution of the Mn-N distances in the photoexcited tetragonal (PT) phase excited at a fluence of 36 mJ.cm^{-2}, and of the photoinduced cubic (PIC) phase fraction x_{PIC}. The associated error bars represent the standard deviation of the Rietveld fit for each delay.

Supplementary Discussion 1. Landau model for coupled charge-transfer and symmetry-breaking

We have previously shown[1,6,7] that the simplest symmetry-adapted Landau potential to be used to describe coupled charge transfer (CT measured through the order parameter q) and symmetry breaking (SB measured through the order parameter η) takes the following form:

$$G(q,\eta) = \frac{1}{2}a\eta^2 + \frac{1}{3}b\eta^3 + \frac{1}{4}c\eta^4 + Aq + \frac{1}{2}Bq^2 + \frac{1}{4}Cq^4 + \frac{1}{2}C_s^0 v_s^2 + \lambda_\eta v_s \eta^2 + \lambda_q v_s\left(\frac{1-q}{2}\right) \qquad (4)$$

The η^2, η^3, η^4 terms describe the usual cubic →tetragonal Landau symmetry breaking potential, with $a = a_0(T - T_{\mathrm{SB}})$ and $c > 0$ for stability.[8] a changes sign at the symmetry-breaking temperature T_{SB}, which stabilizes cubic symmetry ($\eta = 0$) above T_{SB} and tetragonal symmetry ($\eta \neq 0$) below. Similarly, the q, q^2, q^4 terms describe the CT conversion from LT $\mathrm{Mn^{III}Fe^{II}}$ ($q = -1$) to HT $\mathrm{Mn^{II}Fe^{III}}$ ($q = 1$). In this model, a key point is to consider the elastic contributions to the potential: the elastic energy $\frac{1}{2}C_s^0 v_s^2$ related to the volume strain v_s through the elastic constant C_s^0, as well as the elastic coupling to v_s of the SB ferroelastic distortion ($\lambda_\eta v_s \eta^2$) and the CT conversion ($\lambda_q v_s(\frac{1-q}{2})$ is taken as reference in HT state). The equilibrium v_s minimizing $G(q,\eta)$ includes contributions from both CT and SB[1,6,7]:

$$v_s = -\frac{\left[\lambda_q\left(\frac{1-q}{2}\right) + \lambda_\eta \eta^2\right]}{C_s^0} = v_{\mathrm{CT}} + v_{\mathrm{SB}} \qquad (5)$$

with $v_s = 0$ in the HT phase ($q = 1, \eta = 0$) and $v_s < 0$ in the LT phase ($q = -1, \eta \neq 0$). We have shown that substituting v_s in equation (4) leads to the following Landau expansion[1]:

$$G(q,\eta) = \frac{1}{2}a\eta^2 + \frac{1}{3}b\eta^3 + \frac{1}{4}c\eta^4 + Aq + \frac{1}{2}Bq^2 + \frac{1}{4}Cq^4 + Dq\eta^2 \qquad (6)$$

where $D > 0$ stabilizes the cubic HT phase ($q = 1, \eta = 0$) or the LT tetragonal phase ($q = -1, \eta \neq 0$).

In this paper we focus our attention on the symmetry breaking part of equation (4) corresponding to the Landau potential for the cubic →tetragonal ferroelastic phase transition:[9-11]

$$G(\eta) = \frac{1}{2}a\eta^2 + \lambda_\eta v_s \eta^2 + \frac{1}{3}b\eta^3 + \frac{1}{4}c\eta^4 = \frac{1}{2}a'\eta^2 + \frac{1}{3}b\eta^3 + \frac{1}{4}c\eta^4 \qquad (7)$$

where the η^2 coefficient $a' = a_0(T - T_{SB}) + \lambda_\eta v_s$ includes the elastic coupling to v_s of the SB ferroelastic distortion. Inside the thermal hysteresis the high-volume cubic phase ($\eta_{HT} = 0, v_s = 0$) and low-volume tetragonal phase ($\eta_{LT} \neq 0$, $v_s < 0$) can be equally stable ($\lambda_\eta v_s \eta^2 < 0$). This is shown by the $G(\eta)$ curve in Fig. 4, left, which corresponds to the blue curve in Supplementary Fig. 10. The less-bonding CT $\mathrm{Mn^{II}Fe^{III}}$ polarons, photoinduced within 200 fs,[12] result in a photoinduced volume expansion ($\Delta v > 0$), and therefore in an internal pressure, because they are long-lived (≃10 µs) compared to the elastic equilibration timescale of the lattice. The associated volume expansions Δv is destabilizing the tetragonal lattice, through the elastic coupling term ($\propto \Delta v \eta^2 > 0$). For low fluence, corresponding to the polaronic regime (Fig. 4a and light blue curve in Supplementary Fig. 10), the small volume expansion δv increases the coupling term ($\propto \delta v \eta^2$), which reduces the ferroelastic distortion in the equilibrated PT state ($\eta_{\mathrm{PT}} < \eta_{\mathrm{LT}}$). For high fluence, corresponding to the PIPT regime (Fig. 4b and purple curve in Supplementary Fig. 10), the larger volume expansion ΔV increases the coupling term ($\propto \Delta V \eta^2$), which fully destabilizes the tetragonal lattice towards the higher volume cubic symmetry ($\eta = 0$).

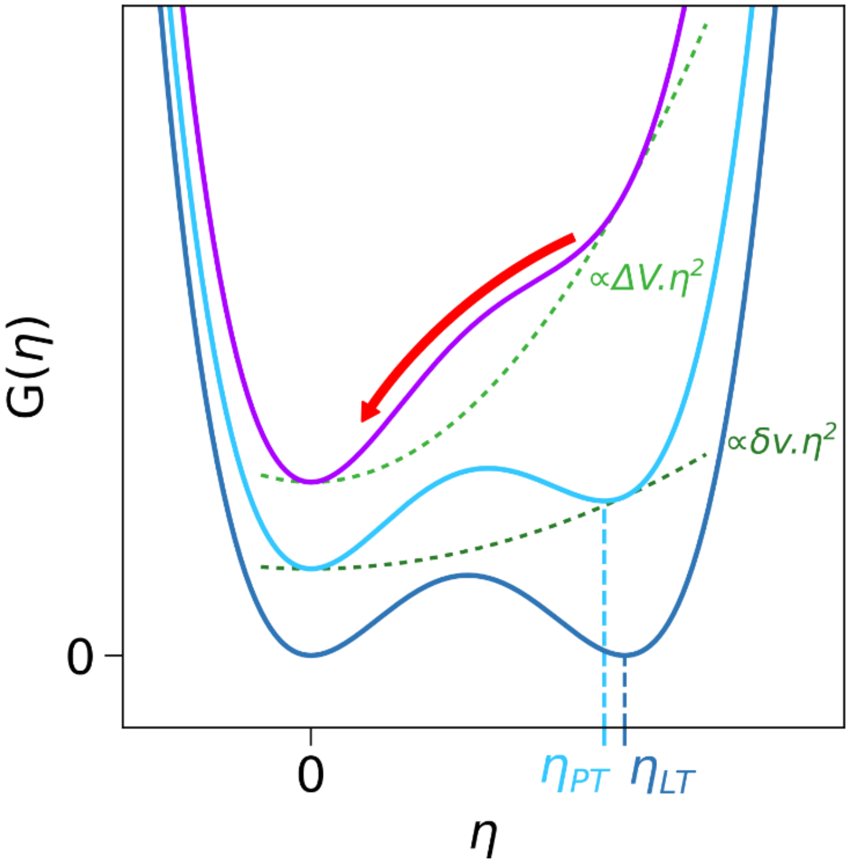

Supplementary Fig. 10. **Evolution of the Landau symmetry-breaking potential**. At equilibrium (blue) the cubic high-temperature (HT, $\eta = 0$) and tetragonal low-temperature (LT, $\eta_{\mathrm{LT}} \neq 0$) phases are equally stable. A weak photoinduced volume expansion (δv) modifies the potential (light blue), due to the elastic coupling cost ($\propto \delta v \eta^2$, represented as a dashed green line), leading to a reduced ferroelastic distortion in the photoexcited tetragonal phase (PT, $\eta_{\mathrm{PT}} < \eta_{LT}$). Larger volume expansion (ΔV) destabilizes the tetragonal lattice towards the cubic lattice (purple).

Supplementary References

1. Azzolina, G. *et al.* Landau theory for non-symmetry-breaking electronic instability coupled to symmetry-breaking order parameter applied to Prussian blue analog. *Physical Review B* **102**, 134104, (2020).
2. Cammarata, M. *et al.* Chopper system for time resolved experiments with synchrotron radiation. *Rev Sci Instrum* **80**, 015101, (2009).
3. Ohkoshi, S., Nuida, T., Matsuda, T., Tokoro, H. & Hashimoto, K. The dielectric constant in a thermal phase transition magnetic material composed of rubidium manganese hexacyanoferrate observed by spectroscopic ellipsometry. *Journal of Materials Chemistry* **15**, 3291, (2005).
4. Ashiotis, G. *et al.* The fast azimuthal integration Python library: pyFAI. *Journal of Applied Crystallography* **48**, 510-519, (2015).
5. Coelho, A. TOPAS and TOPAS-Academic: an optimization program integrating computer algebra and crystallographic objects written in C++. *Journal of Applied Crystallography* **51**, 210-218, (2018).
6. Collet, E., Azzolina, G., Jeftić, J. & Lemée-Cailleau, M.-H. Coupled spin cross-over and ferroelasticity: revisiting the prototype [Fe(ptz)6](BF4)2 material. *Advances in Physics: X* **8**, 2161936, (2023).
7. Collet, E. & Azzolina, G. Coupling and decoupling of spin crossover and ferroelastic distortion: Unsymmetric hysteresis loop, phase diagram, and sequence of phases. *Physical Review Materials* **5**, 044401, (2021).
8. Salje, E. K. *Phase Transitions in Ferroelastic and Co-elastic Crystals*. (Cambridge University Press, 1991).
9. Salje, E. K. & Carpenter, M. A. Linear-quadratic order parameter coupling and multiferroic phase transitions. *J Phys Condens Matter* **23**, 462202, (2011).
10. Carpenter, M. A., Salje, E. K. H. & Graeme-Barber, A. Spontaneous strain as a determinant of thermodynamic properties for phase transitions in minerals. *Eur. J. Mineral.* **10**, 621-691, (1998).
11. Carpenter, M. A. & Salje, E. K. Elastic anomalies in minerals due to structural phase transitions. *Eur. J. Mineral.* **10**, 693, (1998).
12. Azzolina, G. *et al.* Exploring Ultrafast Photoswitching Pathways in RbMnFe Prussian Blue Analogue. *Angewandte Chemie International Edition* **60**, 23267-23273, (2021).
